# Supplementary material for: Are carbon emissions trading and green financial instruments synergistic? -Comprehensive quantitative research based on content analysis
Source: PLoS One. 2024 Mar 7;19(3):e0298601. doi: 10.1371/journal.pone.0298601 (PMC10919601; doi:10.1371/journal.pone.0298601)
Supplement: S1 Data — (ZIP) [file pone.0298601.s001.zip › Data/地级市Z_F工作报告.zip!/║╙─╧/σo╤⌠╩╨/└┤╫╘╣1⁄2╓┌║┼í╛┬φ┐╦╩2╛▌═°í┐_2012.docx]

各位代表：

　　现在，我代表市人民政府，向大会作政府工作报告，请予审议，并请政协各位委员提出意见。

　　一、2011年工作回顾

　　 2011年是濮阳发展历程中具有里程碑意义的一年。一年来，在省委、省政府和市委的正确领导下，全市上下深入贯彻落实科学发展观，准确把握“四个重在”实践要领，以持续开展“一创双优”活动为动力，抢抓中原经济区建设历史机遇，坚持“4321”发展思路，凝心聚力求发展，奋发后起图赶超，真抓实干惠民生，濮阳呈现出科学发展、务实发展、赶超发展的好趋势、好态势、好气势，多项工作在全省有了新的进步，多项经济指标在全省上升了新的位次，社会事业有了新发展、新起色、新提升，城乡面貌日新月异、焕然一新，圆满完成了市六届人大三次会议确定的目标任务，实现了“十二五”规划良好开局。可以说，经过全市人民的共同努力，濮阳正在发生历史性的可喜变化，濮阳正在树立蒸蒸日上的崭新形象，濮阳正在迈入赶超发展的新阶段，濮阳人民得到了更多实惠，自豪感、幸福感不断增强。

　　―――经济形势持续向好。预计全市生产总值完成943亿元，增长11.8%，增速居全省第10位，较去年同期前移5个位次；规模以上工业增加值541亿元，增长16.5%，居全省第16位，与去年同期持平；财政一般预算收入39亿元，增长29.3%，居全省第5位，前移8个位次；社会消费品零售总额274亿元，增长18.8%，居全省第3位，前移11个位次。

　　―――发展后劲持续增强。全市固定资产投资581.9亿元，增长28%，居全省第2位，前移12个位次。

　　―――人民生活持续改善。城镇居民人均可支配收入17076元，增长12.8%，居全省第14位，前移2个位次；农民人均纯收入6092元，增长20%，居全省第2位，前移2个位次。

　　―――社会大局和谐稳定。社会保障体系进一步完善，公共 卫生体系建设得到加强，安全保障水平继续提高，信访、社会治安、民族宗教等工作取得新成绩，社会大局保持和谐稳定。

　　一年来的主要工作做法和成效是：(一)产业支撑能力明显提升。强力实施工业强市战略，围绕石油化工、煤化工、盐化工“三化”融合链接发展，做大做强石油化工产业，做强做优传统产业，加快发展高新技术产业和先进制造业。三次产业比例由2010年的13.9∶66.5∶19.6调整为13.5∶67.8∶18.7，工业对濮阳发展的支撑作用不断增强。油气化工等优势产业完成增加值460亿元，占全市规模以上工业增加值的85%。规模以上工业企业实现销售收入1850亿元，增长30.5%；实现利润150亿元，增长40.1%；实现税金91亿元，增长40.2%。积极推动现代服务业发展，中心城区现代服务业园区入驻企业196家，实现营业收入25亿元。优化金融环境，处置不良贷款13.2亿元。市商业银行濮阳县、南乐县支行挂牌营业，台前县农信社改制为农村商业银行。《水秀III》商演取得成功；龙湖商务广场开工建设；东北庄杂技之乡文化遗产保护与开发等项目顺利推进。全年接待游客354.9万人次，实现旅游收入5亿元。

　　(二)重点项目建设实现重大突破。建立健全了项目推进机制，累计召开重点项目周调度会31次，研究解决了80个项目320项难点问题。实施省定重点项目16个。实施市定重点项目246个，亿元以上的项目162个，完成投资388亿元，较2010年增加255亿元，增长192%。中原石化MTO、永龙化工100万吨真空盐等141个项目建成投产。文96储气库、永金化工20万吨乙二醇等89个项目加快推进。利用八、九、十3个月集中谋划包装项目，初步谋划项目1111个，总投资5889.3亿元，是“十二五”规划谋划项目的两倍。其中，集中包装重大项目324个，总投资2619亿元。确定2012年第一批市重点项目252个，总投资1461.3亿元，年度计划投资506.9亿元；已申报第一批省重点项目80个，总投资1157.8亿元，年度计划投资	272.6亿元。

　　(三)产业集聚区竞相发展。围绕打造科学发展载体，深入开展了“产业集聚区建设提升年”活动。8个省定产业集聚区完成投资233亿元，其中基础设施投资40亿元。建成标准厂房15万平方米。完成融资17.6亿元。新入驻超亿元项目97个。主营业务收入500亿元，安置就业4.2万人。投资30亿元的天能循环经济产业园、投资28亿元的温州创业园、投资20亿元的康益医疗器械加工、投资15亿元的丰利石化扩能改造、投资4亿元的好风景家私、投资1.5亿元的众鑫羽绒等一批产业项目落户产业集聚区，产业集聚效应日益显现。

　　(四)改革开放深入推进。积极参加国家和省举办的招商活动，大力开展自主招商，签约项目175个，总投资908.45亿元，合同市外资金890亿元。实际利用境外资金1.27亿美元，市外资金实际到位160亿元。加强同央企、省企对接，中原油田石化总厂扩能改造、日照经濮阳至洛阳输油管线等项目进入河南省与中石化补充协议。中石油产业扶贫示范区建设进展顺利。油田改制企业“退城入园”、濮阳石化产业园等油地战略合作项目取得新进展。与华润燃气、国电河南电力、河南投资集团等战略投资者签订合作协议。积极做好外经外贸工作，外经指标位居全省前列。统筹推进各项改革，完成了林钢整体移交、4户市直机关所属企业脱钩改制，公用事业改革、公立医院改革试点等各项改革稳步推进。努力破解土地瓶颈，获建设用地指标1.5万亩。非公有制经济完成增加值566亿元，增长14%，占全市经济总量的60%。

　　(五)城市面貌焕然一新。综合提升建成区，开发建设新城区，加强县城、小城镇、新型农村社区建设，城镇化步伐不断加快。中心城市综合提升成效显著。建立了卫生城市迎检、文明城市创建和中心城市综合提升“三城联动”机制，再次被命名为国家卫生城市，获得全国文明城市提名资格。华龙区尧当村、开发区娄店村等8个城中村改造项目开工。

　　建设路等12条道路绿化景观改造提升基本完成。

　　石化路西延、古城路西延建成通车。市污水处理厂二期工程顺利完工，第二污水处理厂开工建设。背街小巷、垃圾站、公厕等改造扎实推进。全市在建房屋面积突破1000万平方米。新区建设扎实推进。新区起步区 “六纵四横”路网工程建设完成投资6.8亿元。引黄调蓄水库前期工作已经完成，开工在即。

　　城镇建设取得新成绩。南乐县等县城综合提升效果明显。8个乡撤乡建镇获省批复。完成了22个新型农村社区项目规划，15个项目开工建设。全市城镇化率33.46%，较2010年增加2个百分点。综合交通体系不断完善。晋豫鲁铁路通道濮阳段加快推进；濮范高速基本建成，德商高速范县段开工建设；濮清快道、S212金堤河桥至庆祖段改建工程建成通车，G106濮范高速至黄河桥段改建工程、S101濮范高速至台前县城段改建工程开工建设。

　　(六)“三农”工作取得新成绩。突出抓好农业农村五项重点工作， “三农”工作全面推进。粮食总产253.7万吨，连续8年实现增产。有效灌溉面积达到332万亩。植树1720万株。林下经济达到15.3万亩。新建、扩建设施农业园区80个，设施农业面积达到30.9万亩。畜牧业总产值84亿元。

　　181家市级以上农业产业化龙头企业实现销售收入126.1亿元，利税13.9亿元，带动农户37.8万户。金堤河二期治理工程基本完工。新改建农村公路130公里。新增沼气用户6000户，新建大中型沼气工程16处。解决了30万人的饮水安全问题。

　　免除了农业水费。完成了30个市级重点示范村基础设施建设任务。完成了61个贫困村整村推进任务。农村劳动力转移72.3万人次，实现劳务收入69.4亿元。

　　(七)人民群众得到更多实惠。十项民生工程年度目标任务基本完成。就业再就业取得新成绩。城乡低保应保尽保。实施了惠民殡葬政策。企业养老保险、城乡居民社会养老保险、失业保险、城镇职工医疗保险覆盖面不断扩大，失业保险、城镇职工医疗保险实现市级统筹。新农合参合率98.96%。公共卫生服务体系建设得到加强。国家基本药物制度稳步实施。筹措校安工程专项资金7.76亿元，开工项目466个。保障性安居工程完成年度任务。住房公积金归集覆盖面超过全省平均水平。科技、教育事业全面发展，公共文化服务体系日益完善，人口计生、统计、体育、发展研究、工商、质监、残疾人工作、市场发展、机关事务管理、民族宗教、史志、地震、气象、人防、保险、邮政、通信等各项工作取得了新成绩。大力开展国防教育，扎实做好双拥工作。驻濮解放军和武警、消防部队为地方发展和稳定作出了积极贡献。

　　(八)政府自身建设切实加强。持续开展 “一创双优”活动，各级政府行政效能和服务水平进一步提升。法治政府建设取得重大进展。执法监督等工作成效显著，我市被国务院法制办评为全国行政复议工作先进单位。自觉接受人大及其常委会监督，支持政协参政议政。认真听取各民主党派、工商联、无党派人士的意见，支持工会、共青团、妇联等人民团体开展工作。深化监察、审计监督，政府廉政建设取得新成效。加强民主法制建设，第七届村级组织换届选举进展顺利， “五五”普法和依法治理取得新成绩。加强精神文明建设，公民道德水平和城乡文明程度不断提升。

　　濮阳经济社会发展的成效得到了省委、省政府充分肯定。

　　卢展工书记来我市调研时指出“濮阳发展有一个好的趋势、一个好的态势、一个好的气势”，评价 “濮阳发展的思路更加清晰了，作风更加扎实了，精神状态更加振作了，濮阳的发展很有希望”。郭庚茂省长莅濮调研时评价 “濮阳后起有望，振兴有望”，对濮阳发展提出了 “重在持续，保持韧劲，坚持不懈，乘势而上”的新要求。省四大班子领导和众多省直部门负责同志相继莅濮调研，纷纷表示看好濮阳、支持濮阳。我们相信，只要鼓足干劲，保持韧劲，持续求进，奋力赶超，濮阳一定能够实现大发展、大跨越，人民群众一定能够得到更多实惠。

　　上述成绩的取得，是省委、省政府和市委正确领导的结果，是人大、政协和社会各界大力支持的结果，是全市人民团结奋斗的结果。在此，我代表市人民政府，向工作在全市各条战线的工人、农民、知识分子、干部、人民解放军指战员、武警消防官兵、公安民警以及所有关心、支持濮阳发展的各界人士致以崇高的敬意，表示衷心的感谢！回顾一年来的工作，我们的主要经验和体会是：(一)创新思想观念、理清发展思路是实现赶超发展的总开关。一年来，我们持续开展 “一创双优”活动，抓住了关键、切中了要害、找到了症结、选准了路径，进一步激发了发展活力，凝聚了发展合力。我们立足中原经济区建设全局，准确研判形势，高点定位谋发展，变 “边缘”为 “前沿”，变“交界”为 “交会”，变 “落后”为 “赶超”，理出了 “4321”发展思路： “4”是牢牢把握建设石油化工、煤化工、盐化工“三化”融合链接示范区、中原经济区重要出海通道、中原经济区与环渤海经济圈衔接融合的前沿、省际交会区域性中心城市的四个战略定位； “3”是走新型工业化、新型城镇化、新型农业现代化 “三化”协调发展的路子； “2”是突出新型工业化、新型城镇化 “两轮驱动”； “1”是奋力实现 “二三五”这一赶超目标。明晰了 “做大做强石油化工产业、做强做优传统产业、加快发展高新技术产业和先进制造业”的工业强市思路、 “综合提升建成区、加快开发建设新区”的城市建设思路和 “坚持不懈抓好农业农村重点工作”的农业现代化思路。形成了赶超发展、改革开放、统筹协调、改善民生四大发展理念。树立了财政保民生保运行、建设靠融资，抓城建就是抓发展，以市场理念经营城市，以改革的办法破解难题，以项目为抓手推动工作等新观念。新的思路、新的理念和新的观念，激发了新动力，明确了新方向，引领了新发展。

　　(二)优化干部作风、提升工作标准是实现赶超发展的强劲动力。一年来，我们突出优化干部作风，围绕科学发展、务实发展、赶超发展，对照 “十破十立”、 “八弃八扬”，转作风、树形象，弘扬 “三李”精神，倡导濮阳干部文化，坚持“三个带头”， “三项要求”得到贯彻， “六超”精神得到发扬， “六新要求”化为行动。同时，创新体制机制，成立督查局、城管局、投资办，整合力量，强化监管督导，并出台了分包负责、台账管理、项目调度、观摩考核、奖惩激励等40多项政策和机制，极大激发了广大干部干事创业的热情。现在，从城市到乡村、从机关到基层，广大干部的群众观念、服务意识不断增强，工作节奏明显加快、工作标准明显提高，工作作风明显转变。早餐会、大锅灶等办法将问题解决在一线；进农户、解民忧、顺民意等措施实现了和谐拆迁；上门服务、登门招商，促进了产业发展；温州创业园项目10天办结落地手续，创造了 “濮阳速度”。明争暗赛、你追我赶、百舸争流的良好局面正在形成。

　　(三)优化发展环境、改善对外形象是实现赶超发展的根本保障。一年来，我们坚持以优化发展环境提升综合竞争力。

　　强化转办交办、督查督办、效能监察，加大对影响、破坏发展环境行为的查处力度。软、懒、散现象得到有效治理，一批影 响较大、久拖未决的问题得到妥善解决，以权谋私、吃拿卡要等行政乱作为的案件被严肃查处，敲诈勒索、封门堵路等损害发展环境的行为受到严厉打击。开展 “优化金融环境、打造诚信濮阳”专项活动，有效改善了金融发展环境。好环境形成了新优势。越来越多的客商云集濮阳，投资濮阳。天能循环经济产业园、南方家私中部产业基地等一批重大项目引进落地。濮阳的形象越来越好，濮阳的吸引力越来越强。

　　各位代表：在肯定成绩的同时，我们也清醒地认识到，我市经济社会发展中还存在不少问题和薄弱环节：一是虽然主要经济指标增幅在全省位次有所提升，濮阳正在发生可喜变化，但并没有发生根本性变化，规模以上工业增加值增速、城镇居民可支配收入增速、外贸出口总额增速、实际利用外资增速等指标仍然低于全省平均水平，赶超发展的任务依然十分艰巨。二是工业支撑发展的能力不强，城镇化水平较低，县域经济落后，农业农村基础薄弱，加快转变经济发展方式的任务繁重。三是重大项目规模较小，质量不高，落地不快，发展后劲需要进一步增强。

　　四是开放水平不高，招商引资工作需要进一步提升。改革力度不够，体制机制不活等问题突出。五是少数地方和部门工作标准不高、节奏缓慢，不善做群众工作，缺乏运作事、办成事的能力。对于上述问题，我们要予以高度重视，认真加以解决。

　　二、2012年政府工作总体要求和奋斗目标

　　2012年是我市立足中原经济区建设大局，实现 “二三五”赶超目标的关键之年。总体看，我们面临的形势依然复杂。一方面，我们仍面临着严峻挑战和压力。从国际看，国际金融危机的影响短期内难以消除，世界经济复苏的长期性、艰巨性和复杂性更加凸显。从国内看，我国经济发展中不平衡、不协调、不可持续的矛盾和问题仍然突出，经济增速回落与物价仍处高位相互交织。从区域看，省内和周边各地市百舸争流，竞相发展，区域竞争日趋激烈。再加上我市经济发展对资源的依赖较重，产业结构不合理的矛盾仍然突出，发展压力越来越大。另一方面，我们面临着难得的发展机遇。从外部环境看，经济全球化趋势不可逆转，国外和沿海地区企业向中西部加快转移的趋势没有改变。我国仍处在重要战略机遇期，中央继续实施积极的财政政策和稳健的货币政策，继续加大对 “三农”、社会事业等领域的投入。中原经济区上升为国家战略， 《国务院关于支持河南省加快建设中原经济区的指导意见》、 《河南省建设中原经济区纲要》多处明确支持濮阳，我市被列为国家资源型城市转型试点，濮阳发展将会获得更多的政策、项目和资金支持。尤其是，通过持续开展 “一创双优”活动，濮阳经济社会发展的好态势、好趋势和好气势进一步形成，具备了科学发展、务实发展、赶超发展的基础和条件。为此，我们要切实增强机遇意识和忧患意识，化挑战为机遇，牢牢把握各项工作的主动权。

　　今年政府工作的总体要求是：深入贯彻落实科学发展观，认真落实中央、省委、市委经济工作会议精神，以 “持续求进、奋力赶超”为总基调，以持续开展 “一创双优”活动为动力，抢抓中原经济区建设重大机遇，坚持 “4321”发展思路，突出实施 “工业强市”战略和新型城镇化引领，着力推动产业集聚区提质增效，着力推动项目建设，着力提升城乡统筹发展水平，着力推动改革开放，着力改善民生和维护社会稳定，全面推进经济建设、政治建设、文化建设、社会建设和生态文明建设，努力使我市主要经济指标赶上或超过全省平均水平，加快建设富裕和谐美丽新濮阳。

　　今年经济社会发展主要预期目标是：生产总值增长11%以上，规模突破1000亿元；地方财政一般预算收入增长15%；规模以上工业增加值增长18%；固定资产投资增长24%；社会消费品零售总额增长18%；外贸出口总额增长15%，实际利用外资增长25%；城镇居民人均可支配收入增长10%，农民人均纯收入增长10%；居民消费价格涨幅控制在4%左右；人口自然增长率控制在6‰以下；城镇登记失业率控制在4.5%以内；城镇化率提高3个百分点；节能减排完成省定目标。

　　三、2012年政府工作主要任务

　　(一)坚定不移实施工业强市战略，强化工业经济对发展的支撑作用。一是实施一批重大工业项目。年内实施工业重点项目104个，总投资483.9亿元，年度计划投资232.2亿元。

　　建成投产永金化工20万吨乙二醇、恒润石化碳四综合利用一期工程、天能循环经济产业园一期工程、康益医疗器械加工、温州创业园高新医疗器械一期工程、上海伽思一期工程、全友家私清丰县产业园等项目；开工建设恒润石化碳四综合利用二期工程、中原大化碳酸二甲酯 (DMC)、宏业精细化工产业园、双虎家具清丰产业园、濮阳电子信息配套产业园、永乐生物产业园等项目；做好中原油田石化总厂扩能改造、中原石化改扩建、龙丰纸业二期等项目前期工作。二是培育壮大一批龙头企业。支持中原油田、中原石化、国电集团、河南煤化集团、河南投资集团等驻濮大企业做大做强。推动濮耐、恒润石化、宏业化工、颖泰化工等地方骨干企业发展壮大。扶持惠成化工、林氏化学等拥有自主知识产权的企业增强核心竞争力。

　　三是加快打造发展载体。8个产业集聚区完成投资320亿元，其中，工业项目投资280亿元，实现营业收入700亿元；基础设施及其他配套设施投资40亿元。强基础、促配套。加快集聚区对外连接通道、环保、供电、生活服务等基础设施以及职工培训、物流仓储、检验检测等配套设施项目建设。统一规划建设生活配套功能服务区，生产区内禁止建设职工公寓。各产业集聚区发展规划展览馆3月底前建成投用。引龙头、促集聚。围绕产业定位，引进一批带动力强、辐射力大的龙头型、基地型项目和关联度高的配套型项目，推进产业集聚发展和产业链条延伸。抓保控、促集约。集中土地指标、环境容量等资源要素，重点支持高投资强度、高技术含量、高附加值的项目。投资强度低于234万元/亩省定标准的入区工业项目，不予单独供地，进入标准化厂房生产。找对手、促发展。创新产业集聚区观摩考评机制，完善基础设施、入驻项目、项目质量、投产达效等考评办法。开展各县区 “对手赛、联手赛”活动，促使产业集聚区竞相发展。同时，研究出台开发区、工业园区发展扶持政策，促其实现跨越发展。四是助推企业上市。

　　加快蔚林化工、惠成电子、中拓管道、绿能高科、贝英数控、泓天威药业等企业上市步伐，再筛选10家上市和股权交易后备企业。五是推动自主创新。抓好创新平台建设，加快建设科技企业孵化器。扶持4家企业组建市级工程技术研究中心，新培育1家省级工程技术研究中心。强化产学研合作，组织实施产学研合作重点项目10项。建设上海国家技术转移联盟濮阳工作站。新签订全面合作协议高校院所2家。实施质量立市战略，建成投用市产品质量综合检测实验楼暨国家电光源质检中心，做好品牌创建工作。六是加强节能减排。实行强度和总量 双控制，落实节能降耗综合工作方案，完善评价考核奖惩制度，加强产业准入管理，淘汰落后产能。开展企业节能低碳、绿色节能建筑等节能专项行动。抓好能源、化工、造纸等重点行业节能减排。启动机动车尾气治理工作。实施污染综合整治，搞好境内河流沿线环境保护。

　　(二)坚定不移推进中心城市综合提升和新区建设，加快新型城镇化步伐。年内实施城建重点项目69个，总投资426.9亿元，年度计划投资118.5亿元。全市完成建房面积1300万平方米，其中，城区达到650万平方米。一是强力推进中心城市综合提升。推进建成区路网项目。竣工长庆路南延、石化中路、石化东路、胜利东路、玉门路、帝丘路、惠民路等7条道路。开工建设站前路西延、文化路北延、任丘路西延、南海路东延、马颊河滨河西路及滨河东路、玉门路北延、苏北路西延、大庆路南延拓宽改造、中原路东段改造10条道路。完成开州路、中原路、濮上路3条样板路改造。完成振兴路、昆吾路、金堤路、人民路、京开大道5条道路部分路段，以及中原路与京开大道转盘改造工程。推进改造提升项目。科学规划城市集贸市场。对城区市场施行统一监督管理。强力推进中原市场、劳动市场拆迁改造，加快大庆市场建设进度。加快推进中心城区城中村、旧城区、集中连片棚户区改造和城郊村并入城市。加快尧当、娄店等已开工城中村改造项目进度，完成回迁房主体建设。市城区23个城中村改造项目全部开工建设。完成市城区所有跨路电线电缆 “上改下”整治任务，完成开州路、中原路、濮上路顺路电线电缆入地。推进绿化美化亮化项目。完成高速引路风景林带等6个绿化提升项目，新建2处以上大型街头绿地，城区新增绿地18万平方米以上。完善和维护4大夜景片区和9大主干道亮化景观。完成建成区主干道两侧主要建筑、大型公共建筑和公园、广场亮化工程。同时，强化 “六乱”治理。加快推进数字城管工作。建立国家卫生城市长效管理机制，开展城乡卫生整治行动。开展文明单位与园林单位联创活动。做好国家园林城市复查准备。二是加快濮阳新区建设。坚持规划先行，基础设施跟进，项目统筹开工，实现“三年见成效、五年成规模”。加快规划编制。尽快完成 《濮阳新区建设总体方案》报批。做好新区相关规划及城市设计方案的征集与组织评审。完成新区行政文化核心区10平方公里和职教园区5平方公里控规及城市设计。加快基础设施建设。6月底前完成开州路北延、濮上路北延、昆吾路北延、卫河路北延、历山路北延、濮水路北延、五一路西延和开德路、绿城路改造9条已开工道路续建任务。年底前完成内环东路、卫都路等5条道路建设。加快引黄调蓄水库建设。推进供水、燃气、供热管网等公用设施建设。推进商检海关商务综合办公楼、移动公司通信生产楼等项目建设。三是统筹推进城镇建设。加大县城、小城镇提升和新型农村社区建设力度。做优做强县城。

　　推进濮阳县、市城区、清丰县一体化进程，率先在道路、污水、供暖等基础设施上实现联通。将濮阳县城、清丰县马庄桥镇纳入城市管理考核范围。支持南乐县、范县、台前县内涵式发展，强化产城一体化。加大县城改造、建设、管理力度，明确年度目标任务，建立详细工作台账。从今年开始，将县城建设情况与产业集聚区建设情况一并观摩。发展壮大小城镇。推进城镇供排水、垃圾污水处理等基础设施建设。通过专业园区建设，提高柳屯镇、文留镇、濮城镇等小城镇的承载能力。完成4个乡撤乡建镇。完成台前县城、清丰县马庄桥镇气化工程项目，加快公用基础设施向农村延伸。推进新型农村社区建设。以产业集聚区和县城、小城镇的村庄整合为重点，积极稳妥推进新型农村社区建设。市财政拿出5000万元，实行 “以奖代补”，集中支持开发区、工业园区内的新型农村社区建设。

　　(三)坚定不移加快基础设施建设，切实改善经济社会发展基础条件。一是推进综合交通体系建设。铁路方面，加快晋豫鲁铁路通道濮阳段建设。积极推进郑濮济客专前期工作。高速公路方面，加快德商高速范县段建设。开工建设南林高速东延至豫鲁省界段。争取年内开工范台梁高速。做好濮阳至东明高速前期工作。干线公路方面，完成G106濮范高速至黄河桥段改建工程、S101市城区段改建工程、S101濮范高速至台前县城段改建工程。开工建设G106濮范高速至豫冀省界段改建工程。农村公路方面，重点抓好偏僻、贫困乡镇农村公路建设，投资1.6亿元，新改建县乡公路150公里，改造危桥500延米。筹措养护资金3000万元，提高农村公路养护质量和水平。二是推进水利设施建设。结合濮阳水系规划和引黄调蓄水库建设，全面实施濮水河、马颊河、第三濮清南生态环境综合治理，打造水景观、发展水经济、培育水文化。开工建设引黄入冀补淀工程濮阳段、南水北调濮阳支线工程及配套水厂管网。实施渠村、南小堤、彭楼三个大型灌区续建配套与节水改造。开工建设邢庙、满庄两个中型灌区续建配套与节水改造。

　　对永顺沟、潴泷河下段进行治理。加快实施小型农田水利重点县建设。推进清丰县、南乐杏元引黄调蓄工程前期工作。三是推进能源设施建设。建成投用文96储气库。推进文23战略储气库、日照经濮阳至洛阳输油管线、濮阳煤炭储配中心等项目前期工作。开工建设南乐汇通生物质发电项目、濮东500千伏输变电工程。建成投运濮阳工业园区背压机组。加快推进龙丰热电2×60万千瓦机组项目，争取年内开工建设。四是完善城市配套设施建设。完成市第二污水处理厂土建工程。

　　开工建设濮阳县污水处理厂、工业园区污水处理厂。完成建筑垃圾处理场扩建。实施濮水公园扩建项目。加快城区污雨水泵站和管网、垃圾中转站、公厕等公共设施建设。开工建设公交绿城综合保养场。建设站前广场。启动飞龙、信达汽车站迁建工作。加快无线城市建设。推动有线电视尽快实现数字化整体转换。

　　(四)坚定不移做好农业农村工作，着力夯实新型农业现代化基础。一是做好农业农村五项重点工作。统筹做好农田水利基本建设、造林绿化、设施农业发展、农村土地综合整治、村容村貌整治五项重点工作，夯实 “三农”工作基础。二是稳定粮食生产。落实粮食生产直接补贴等各项强农惠农政策。按照 “合理规划、综合配套、稳定面积、主攻单产”的原则，推进粮食生产核心区建设，规划建设百亩方、千亩方和万亩方高标准永久性粮田。加强基层农技推广体系和农业信息体系建设。提高农业机械化水平。支持良种繁育和种子产业发展。粮食种植面积稳定在560万亩以上，粮食总产保持在250万吨左右。三是加快调整农业结构。调整种植业结构。设施农业总面积达到38万亩，打造高标准设施农业园区50个、设施农业现代化精品园区15个。争创2个农业部蔬菜标准种植园。加快建设南乐县10万亩水果和3万亩节水莲藕、台前县万亩优质大蒜等高效农业基地，建成范县万亩泥鳅养殖基地、濮阳高效生态农业观光园等项目。调整农村产业结构。新建和改建标准化养殖场区40个。开展 “奶业推进年”活动。完成省定林业生态工程造林18万亩以上，完成市定植树任务2000万株以上，全市林业总产值70亿元。调整农村经济结构。因地制宜发展农村二、三产业。推进农村土地流转，实现规模化经营。

　　提高农业生产的组织化程度，全市农民专业合作经济组织发展 到1700家。发展劳务经济，继续实施 “阳光工程”和 “雨露计划”，转移培训农民工和低收入人口1.2万人。四是推进农业产业化。培育壮大农业产业化龙头企业，年销售收入超亿元的达到25家，超10亿元的达到2家。开工建设投资5.2亿元的豫粮集团濮阳粮食产业园区、投资20亿元的大用集团肉鸡产业化等一批重大项目。五是推进新农村建设。对国道省道沿线、产业集聚区及周边村庄环境进行综合整治提升，重点抓好12个省级、40个市级新农村示范村建设。完成50个贫困村整村推进任务，实现4.2万人脱贫。实施农村沼气工程，新建沼气联户工程20座、新增户用沼气2000户。

　　(五)坚定不移发展文化产业和现代服务业，加快培育新的经济增长点。一是培育壮大文化旅游产业。抓好文化改革发展试验区建设，打造杂技文化和龙文化两张 “名片”。加快推进龙湖商务广场、濮阳国际杂技文化产业园、东北庄杂技之乡文化遗产保护与开发、将军渡黄河游览区、单拐革命旧址综合开发、范县革命烈士纪念馆、市豫剧团排练厅改造等项目建设。组织举办好2012濮阳中华龙文化节。争取把黄河旅游观光带纳入河南省大黄河之旅规划。绿色庄园杂技表演馆完成升级改造。实现接待旅游总人数390万人次以上，旅游收入5.5亿元以上。二是大力发展商贸物流业。推进中心城区现代服务业园区建设，加快银座国际酒店、华阳国际大厦等项目建设，开工建设现代商贸广场、万利财富广场二期、亿丰时代广场、崛起时代广场、联华城市广场、浙江中骄集团南乐商贸城等项目。推进豫北商贸物流园区建设，开工建设丰源省际建材物流园、清丰县家具物流博览中心等项目。推进 “万村千乡市场工程”建设。谋划建设国家级综合能源物流中心。三是加快发展现代金融业。丰富金融市场主体。引进1至2家金融机构。市商业银行实现5县全覆盖。完善政府投融资平台体系，充分发挥建投、水投、经开公司等投融资平台的作用。积极推动银企合作，深化金融机构与企业 “1+1”、 “1+N”帮扶，破解中小企业融资难问题。积极发展农业投资基金和农村信用担保公司。第三季度集中开展税收大检查，进一步规范市场经济秩序。

　　(六)坚定不移扩大对外开放，打造省际交会区域性开放高地。一是抓好招商引资。组织开展好 “长三角”、闽东南等发达地区的4次大规模驻地招商活动，积极承接产业转移。依托产业集聚区，引进承接高成长性产业项目、新兴产业项目和劳动密集型项目。瞄准世界500强、国内500强和行业龙头企业开展深度对接。加强与央企合作，积极引进战略投资者。扩大拜访联系央企范围，由20家扩大到44家。落实油地战略合作协议，加快推进濮阳石化产业园等合作项目。落实市政府与河南投资集团战略合作协议，加快同力水泥二期等项目建设。

　　深入开展 “招商引资项目落地年”活动，完善招商引资考评办法，狠抓合同履约率、项目开工率、资金到位率。从今年起，各县区、开发区、工业园区党政主要负责同志每年各引进并落地1个投资超10亿元的大项目，分管负责同志引进并落地1个投资超5亿元的大项目。市商务局引进并落地5个投资超10亿元的大项目。二是抓好外经外贸。加快外贸公共服务平台建设，发挥国家级石油机械出口基地和省级电光源出口基地的示范作用，提升台前县羽绒制品及服装出口基地等县域产业集群。争取我市获批国家级或省级化工出口基地、羽绒制品及服装出口基地。新增有进出口经营权的企业50家。实施科技兴贸战略和机电产品出口推进计划，使机电和科技产品出口额占全市出口总额的比重达到60%以上。争取在濮阳设立海关办事机构，加快建设商检办事机构，推进日照港驻濮阳口岸业务综合服务平台建设，推动 “无水港”建设取得新突破。支持中原油田等企业扩大承包境外工程，帮助濮耐等企业搞好境外生产经营。

　　(七)坚定不移加强改革创新，增强经济发展的动力和活力。一是深化改革。加大国企改革力度，切实改变我市国企改革进度较慢的现状。完成国有企业改制任务，完成市直机关所属企业脱钩改制工作。妥善解决市甲醇厂、市氯碱厂、市三强纺织厂等企业改革遗留问题。强化国有资产监管，盘活存量资产，搞好资本运营，做大做强国有企业。加快推进市天然气公司、市热力公司、市自来水公司改革。深化医药卫生体制改革，推进基层医疗卫生机构综合改革和公立医院改革，将基本药物制度实施范围扩大到村卫生室。加快市县两级财政预算管理制度改革，集中财力保运行、保民生、保重点项目。加大事业单位人事制度改革力度。完善机关事业单位工资制度。深化农村改革，全面开展农村集体土地确权登记发证，推动集体建设用地使用权有序流转。深化行政管理体制、文化企事业单位、县级供销社体制等各项改革。同时，加大对中小企业特别是小型微型企业的支持力度，加快发展非公有制经济。二是破解瓶颈。坚持用改革创新的办法，破解发展瓶颈制约。土地方面。积极开展城乡建设用地增减挂钩试点，盘活存量土地。加大用地储备和指标争取，储备经营性用地2000亩，完成报批土地13000亩。坚持节约集约用地，提高土地利用效率。实施土地差别化政策，用地指标向产业集聚区倾斜，向符合国家产业政策、科技含量高、附加值高、带动能力强的项目倾斜。资金方面。巩固和深化 “优化金融环境、打造诚信濮阳”活动成效，防范化解金融风险，提升金融机构支持地方建设的积极性。通过采取BT、BOT等模式加大社会融资力度。鼓励支持民间资本投资社会事业。环保方面。处理好节能减排与项目建设之间的关系，做到既保证完成减排任务，又确保大项目、好项目顺利建设。对于关系濮阳发展全局的重大项目，提前介入、全过程跟踪服务和指导监督，确保大项目设立一个、环评审批一个、验收合格一个。三是先行先试。把握资源型城市可持续发展、濮范台扶贫开发综合试验区、国家级现代农业示范区、区域物流枢纽、天然气储备基地等重大项目列入 《国务院关于支持河南省加快建设中原经济区的指导意见》的历史机遇，充分利用国家赋予的先行先试权，主动融入对接，大胆改革创新，积极争取国家、省在土地、资金、环保、产业扶贫等方面的支持，赢得中原经济区建设工作主动权。

　　(八)坚定不移做好保障改善民生工作，努力使人民群众得到更多实惠。一是继续抓好民生工程。农村饮水安全。加大对农村饮水安全工程的财政投入，年内解决50万以上农村人口饮水安全问题，确保2013年全市群众都能吃上安全水，提前三年解决农村饮水安全问题。保障性住房建设。开工建设18116套保障性住房，竣工东方花园3880套经济适用住房。

　　制定和完善并严格执行保障性住房分配、管理、退出等制度和办法，确保工程质量和分配公平。今后，我们建设保障性住房原则上以建设廉租房和公共租赁住房为主。社会保障体系建设。扩大社会保险覆盖面，稳步提高保障水平。扎实推进新农保试点工作，年底前基本实现新农保和城镇居民社会养老保险制度全覆盖。建立健全最低工资标准、职工工资、退休人员基本养老金、城乡低保及农村 “五保”供养标准的正常调整机制。按照省统一部署，落实公务员津贴补贴和事业单位绩效工资制度，继续增加企业退休人员基本养老金。

　　将国有、集体企业“老工伤”人员全部纳入工伤保障范围。实现被征地农民社会保障全覆盖。继续提高新农合筹资标准和农民受益水平。重点抓好高校毕业生、城镇就业困难群体、退役人员和残疾人就业帮扶工作，实现全市城镇新增就业人员7万人，失业人员再就业2万人，应届高校毕业生就业率达到80%，动态消除零就业家庭。不断完善社会福利体系，发展慈善事业，建成流浪未成年人救助保护中心。扩大住房公积金覆盖面，提高住房公积金缴存比例。保持物价总水平基本稳定。教育事业发展。把基础教育特别是义务教育硬件建设作为重点，大力推进建设新学校，改造提升老学校校舍，确保在“十二五”末彻底改变我市基础教育硬件建设的落后状况。年内完成市二实小南教学楼、油田十三中教学楼等8个校安工程项目。建设120所标准化学校。华龙区、开发区在城区各建设一所小学。同时，按照“三集中”的思路，规划引导农村小学向中心村集中，初中向城镇集中，高中向县城集中，推动农村中小学合理布局。完善中小学教师支教交流制度，促进义务教育均衡发展。加快实施学前教育三年行动计划，全市新建幼儿园51所，改扩建89所。大力实施职业教育攻坚计划，完成职业教育园区规划审批，加快推进市委党校迁建、市卫校迁建升专、市杂技学校迁建、市技工学校迁建、市外国语高中迁建等学校入园工作，支持濮阳职业技术学院创建河南省骨干高职院校。二是统筹发展社会事业。健全城乡公共卫生服务网络和疾病防控体系，促进新增卫生资源重点向农村倾斜。加快推进市精神卫生中心、市眼科医院迁建等新建项目和市食品药品检验所实验楼改建、市传染病医院应急病房楼、市人民医院全科医生临床培养基地项目和儿科医疗服务体系项目、市级区域卫生信息平台等项目。实施国家和省免费孕前优生健康检查及中日合作家庭保健服务项目，稳定低生育水平，提高出生人口素质。深入实施文化惠民工程，积极推进公益性文化设施免费开放。加大公共体育基础设施建设，开展全民健身运动。统筹做好外事侨务、残疾人工作、发展研究、机关事务管理、统计、保险、地震、气象、人防、史志、工商、邮政等工作。

　　三是全力维护社会稳定。加强重点行业和重点企业的安全监管，坚决遏制重特大安全事故发生。做好以学校周边环境综合治理和校车安全为主的校园安全管理。开展食品药品安全专项整治。落实领导分包信访案件、定期接访、异地评查等信访工作制度，深入解决信访突出问题。维护民族团结和宗教和睦。创新社会管理，化解社会矛盾。加强社会治安综合治理，推进平安濮阳创建，确保社会大局和谐稳定。四是加强精神文明和民主法制建设。深入开展精神文明创建活动。学习 “三李”精神，倡导濮阳干部文化。完善基层民主建设。开展普法教育和依法治理。充分听取各民主党派、工商联和无党派人士的意见，支持工会、共青团、妇联等人民团体参与社会管理和公共服务。加强国防教育和国防后备力量建设，支持驻濮解放军、武警消防部队、民兵预备役部队建设，开创双拥共建新局面。

　　(九)坚定不移加强政府自身建设，提高保障发展能力。

　　一是打造高效政府。以持续开展“一创双优”活动为动力，扎实推进 “两转两提”，加快政府职能转变，减少和规范行政审批事项，提高行政效能、工作效率和服务水平。推行政府绩效管理和行政问责制度，加强效能监察和行政督查，着力解决行政不作为、慢作为、乱作为等问题，确保令行禁止、政令畅通。下决心精简会议和文件，改进会风文风。二是打造法治政府。全面推进依法行政，提高政府依法管理经济社会事务的能力和水平，加快建设法治政府。建立行政调解机制，依法化解社会矛盾纠纷。认真执行人大及其常委会的决议、决定，依法接受其法律监督和工作监督，主动接受人民政协的民主监督，认真办理人大代表建议和政协委员提案。三是打造阳光政府。以建立和完善财政预算公开制度为重点，扩大政务公开范围和层次。建立健全重大决策听证和重要事项公示制度，提高行政决策的透明度和社会公众参与度。加强政务信息查询平台建设，保障社会公众知情权。建设阳光透明的公共资源交易平台。四是打造廉洁政府。落实党风廉政建设责任制，推进惩治和预防腐败体系建设。加强反腐倡廉宣传教育，强化监察、审计等专门监督，加强对权力运行的制约和监督，严肃查处违法违纪案件，认真解决群众反映强烈的突出问题，确保权力规范运行，保障经济社会发展。匡正换届风气，做好县、乡政府换届工作，使换届工作成为承前启后、推动事业发展的新起点。

　　各位代表：

　　回首去年，濮阳发展成果丰硕，濮阳人民信心百倍；展望未来，濮阳发展前景广阔，濮阳人民豪情满怀。让我们在省委、省政府和市委的坚强领导下，以持续开展 “一创双优”活动为动力，以对濮阳历史和子孙后代高度负责的态度，加压奋进，克难攻坚，努力实现“二三五”赶超目标，加快建设富裕和谐美丽新濮阳。濮阳的明天一定会更加美好！
